# Supplementary material for: Impact of the COVID-19 lockdown in the United Kingdom on adolescent’s time use (CONTRAST study)
Source: PLoS One. 2025 Jan 16;20(1):e0310597. doi: 10.1371/journal.pone.0310597 (PMC11737780; doi:10.1371/journal.pone.0310597)
Supplement: S1 Table — (DOCX) [file pone.0310597.s001.docx]

**Impact of the COVID-19 lockdown in the United Kingdom on adolescent’s time use (CONTRAST study)**

I.Pokhilenko,^1^ E. Frew,^1^ M. Murphy,^2^ M. Pallan^2^

^1^Centre for Economics of Obesity, Institute of Applied Health Research, University of Birmingham

^2^Institute of Applied Health Research, University of Birmingham

## **S1 Table. Recoding of time use variables**

| Initial categories | Final categories (hours) |
| --- | --- |
| Daily activities | |
| 0 = No time | 0 |
| 1 = Up to 30 minutes | 0.25 (15 minutes = quarter of an hour) |
| 2 = Between 30 minutes and 1 hour | 0.75 (45 minutes = 3 quarters of an hour) |
| 3 = 1-3 hours | 2 |
| 4 = 4-6 hours | 5 |
| 5 = More than 6 hours | 7 |
| 6 = Don't know | Excluded |
| Weekly exercise | |
| 0 = never | 0 hours |
| 1 = less than once per week | 0.5 hours |
| 2 = once per week | 1 hour |
| 3 = 2-3 times per week | 2.5 hours |
| 4 = 4-5 times per week | 4.5 hours |
| 5 = more than 5 times per week | 6 hours |
