# Supplementary material for: Impact of the COVID-19 lockdown in the United Kingdom on adolescent’s time use (CONTRAST study)
Source: PLoS One. 2025 Jan 16;20(1):e0310597. doi: 10.1371/journal.pone.0310597 (PMC11737780; doi:10.1371/journal.pone.0310597)
Supplement: S2 Table — (DOCX) [file pone.0310597.s002.docx]

**Impact of the COVID-19 lockdown in the United Kingdom on adolescent’s time use (CONTRAST study)**

I.Pokhilenko,^1^ E. Frew,^1^ M. Murphy,^2^ M. Pallan^2^

^1^Centre for Economics of Obesity, Institute of Applied Health Research, University of Birmingham

^2^Institute of Applied Health Research, University of Birmingham

## **S2 Table. Socioeconomic characteristics of the sample**

| FAS score | | FAS group 1 | | | FAS group 2 | | | FAS group 3 | | |
| --- | --- | --- | --- | --- | --- | --- | --- | --- | --- | --- |
| Experience of food insecurity | | Yes | No | Total  (n, %) | Yes | No | Total  (n, %) | Yes | No | Total  (n, %) |
| FSM eligibility | FSM eligible | 10 | 23 | 33  (15%) | 2 | 9 | 11 (5.6%) | 2 | 7 | 9 (4.5%) |
|  | FSM ineligible | 23 | 146 | 169 (76.8%) | 16 | 158 | 174 (88.3%) | 8 | 167 | 175 (87.9%) |
|  | FSM status not known | 2 | 16 | 18  (8.2%) | 0 | 12 | 12 (6.1%) | 1 | 14 | 15 (7.5%) |
| Total (n, %) | | 35  (15.9%) | 185 (84.1%) | 220 (100%) | 18  (9.1%) | 179 (90.9%) | 197 (100%) | 11 (5.5%) | 188 (94.5%) | 199 (100%) |
| Family affluence score (FAS); free school meal (FSM) | | | | | | | | | | |
