# Supplementary material for: Impact of the COVID-19 lockdown in the United Kingdom on adolescent’s time use (CONTRAST study)
Source: PLoS One. 2025 Jan 16;20(1):e0310597. doi: 10.1371/journal.pone.0310597 (PMC11737780; doi:10.1371/journal.pone.0310597)
Supplement: S3 Table — (DOCX) [file pone.0310597.s003.docx]

**Impact of the COVID-19 lockdown in the United Kingdom on adolescent’s time use (CONTRAST study)**

I.Pokhilenko,^1^ E. Frew,^1^ M. Murphy,^2^ M. Pallan^2^

^1^Centre for Economics of Obesity, Institute of Applied Health Research, University of Birmingham

^2^Institute of Applied Health Research, University of Birmingham

## **S3 Table. Descriptive changes in time use by socioeconomic indicators (in hours, 1 hour = 1)**

| Category of time use |  | FAS | | | FSM eligible | | | Food insecurity | |
| --- | --- | --- | --- | --- | --- | --- | --- | --- | --- |
|  |  | 1 (n=236)  Mean (SD) | 2 (n=203)  Mean (SD) | 3 (n=210)  Mean (SD) | No (n=542)  Mean (SD) | Yes (n=55)  Mean (SD) | Don’t know  (n=48)  Mean (SD) | No (n=555)  Mean (SD) | Yes (n=65)  Mean (SD) |
| School work | Before lockdown | 6.62 (1.20) | 6.48 (1.32) | 6.62 (1.53) | 6.56 (1.48) | 6.78 (1.42) | 6.59 (1.16) | 6.54 (1.42) | 6.92 (1.68) |
|  | During lockdown | 4.15 (2.81) | 4.86 (2.80) | 5.08 (2.70) | 4.75 (2.77) | 3.91 (3.14) | 4.80 (2.48) | 4.69 (2.73) | 4.38 (3.23) |
|  | Mean difference (SD) | **-2.42 (2.59)*** | **-1.66 (2.64)*** | **-1.5 (2.8)*** | **-1.78 (2.59)*** | **-2.83 (3.59)*** | **-1.8 (2.6)*** | **-1.82 (2.64)*** | **-2.41 (3.2)*** |
|  | Results of ANOVA/ unpaired t-test comparing differences across groups | **F(2, 593)=6.78, p=0.001** | | | **1.05 (0.29 – 1.81), p=0.007** | |  | 0.58 (-0.15 – 1.31), p=0.12 | |
| Activities outside of schoolwork that help me learn new knowledge and skills | Before lockdown | 1.37 (1.57) | 1.25 (1.50) | 1.12 (1.21) | 1.25 (1.47) | 1.24 (1.05) | 1.32 (1.40) | 1.21 (1.38) | 1.32 (1.60) |
|  | During lockdown | 1.31 (1.42) | 1.01 (1.06) | 1.27 (1.42) | 1.15 (1.23) | 1.45 (1.74) | 1.54 (1.70) | 1.18 (1.21) | 1.34 (1.81) |
|  | Mean difference (SD) | -0.05 (1.63) | **-0.25 (1.32)*** | 0.1 (1.46) | -0.1 (1.46) | 0.04 (1.4) | 0.23 (1.84) | -0.05 (1.43) | -0.06 (1.95) |
|  | Results of ANOVA/ unpaired t-test comparing differences across groups | F(2, 606)=2.63, p=0.07 | | | -0.14 (-0.56 – 0.28), p=0.51 | |  | 0.12 (-0.38 – 0.40), p=0.95 | |
| Reading | Before lockdown | 0.63 (1.15) | 0.67 (1.10) | 0.51 (0.83) | 0.60 (1.02) | 0.69 (1.25) | 0.58 (0.99) | 0.58 (0.99) | 0.77 (1.39) |
|  | During lockdown | 0.65 (1.30) | 0.66 (1.06) | 0.62 (1.10) | 0.62 (1.11) | 0.60 (1.07) | 0.91 (1.73) | 0.62 (1.10) | 0.81 (1.47) |
|  | Mean difference (SD) | 0.01 (0.86) | 0 (0.73) | **0.11 (0.73)*** | 0.02 (0.7) | -0.07 (0.68) | **0.36 (1.39)*** | 0.04 (0.72) | 0.05 (1.18) |
|  | Results of ANOVA/ unpaired t-test comparing differences across groups | F(2, 617)=1.16, p=0.32 | | | 0.09 (-0.10 – 0.29), p=0.35 | |  | -0.01 (-0.21 – 0.20), p=0.95 | |
| Total screen time | Before lockdown | 4.37 (3.56) | 4.50 (4.06) | 3.96 (3.63) | 4.13 (3.60) | 4.09 (3.14) | 6.10 (5.31) | 4.21 (3.58) | 4.46 (4.67) |
|  | During lockdown | 5.93 (4.33) | 5.94 (4.65) | 5.49 (4.21) | 5.63 (4.31) | 6.23 (4.65) | 7.08 (4.85) | 5.66 (4.21) | 6.60 (5.61) |
|  | Mean difference (SD) | **1.44 (3.79)*** | **1.55 (3.02)*** | **1.56 (2.9)*** | **1.52 (3.15)*** | **1.87 (3.89)*** | **1.05 (3.88)*** | **1.45 (2.98)*** | **2.09 (5.25)*** |
|  | Results of ANOVA/ unpaired t-test comparing differences across groups | F(2, 592)=0.09, p=0.92 | | | -0.35 (-1.33 – 0.62), p=0.47 | |  | -0.64 (-1.52 – 0.24), p=0.15 | |
| Social media | Before lockdown | 1.28 (1.42) | 1.37 (1.57) | 1.51 (1.66) | 1.36 (1.55) | 1.27 (1.32) | 1.78 (1.74) | 1.36 (1.50) | 1.31 (1.69) |
|  | During lockdown | 1.79 (1.89) | 1.81 (1.99) | 1.96 (2.01) | 1.80 (1.96) | 2.01 (1.92) | 2.22 (1.98) | 1.82 (1.94) | 2 (2.01) |
|  | Mean difference (SD) | **0.45 (1.61)*** | **0.48 (1.49)*** | **0.47 (1.56)*** | **0.45 (1.48)*** | **0.64 (1.89)*** | 0.44 (1.91) | **0.45 (1.47)*** | **0.69 (2.13)*** |
|  | Results of ANOVA/ unpaired t-test comparing differences across groups | F(2, 604)=0.02, p=0.98 | | | -0.19 (-0.64 – 0.26), p=0.40 | |  | -0.23 (-0.64 – 0.18), p=0.26 | |
| Watching TV/ Netflix/YouTube/  TikTok | Before lockdown | 1.94 (1.73) | 1.96 (1.73) | 1.59 (1.60) | 1.74 (1.60) | 2.03 (1.89) | 2.65 (2.20) | 1.81 (1.64) | 1.98 (1.99) |
|  | During lockdown | 2.59 (2.13) | 2.55 (2.06) | 2.19 (1.91) | 2.38 (1.99) | 2.55 (2.25) | 3.10 (2.29) | 2.39 (1.99) | 2.81 (2.38) |
|  | Mean difference (SD) | **0.62 (1.94)*** | **0.64 (1.59)*** | **0.61 (1.48)*** | **0.65 (1.66)*** | **0.39 (1.66)*** | 0.47 (1.98) | **0.60 (1.61)*** | **0.80 (2.31)*** |
|  | Results of ANOVA/ unpaired t-test comparing differences across groups | F(2, 610)=0.01, p=0.99 | | | 0.26 (-0.21 – 0.74), p=0.28 | |  | -0.20 (-0.65 – 0.24), p=0.37 | |
| Playing games on a device | Before lockdown | 1.17 (1.70) | 1.21 (1.79) | 0.90 (1.43) | 1.04 (1.58) | 1.02 (1.58) | 1.77 (2.26) | 1.07 (1.61) | 1.27 (1.88) |
|  | During lockdown | 1.53 (2.00) | 1.58 (2.10) | 1.34 (1.92) | 1.43 (1.95) | 1.72 (2.23) | 1.84 (2.32) | 1.45 (1.95) | 1.73 (2.33) |
|  | Mean difference (SD) | **0.36 (1.52)*** | **0.36 (1.28)*** | **0.45 (1.22)*** | **0.38 (1.31)*** | **0.71 (1.65)*** | 0.11 (1.39) | **0.37 (1.23)*** | **0.49 (2.10)*** |
|  | Results of ANOVA/ unpaired t-test comparing differences across groups | F(2, 608)=p=0.76 | | | -0.32 (-0.71 – 0.06), p=0.10 | |  | -0.12 (-0.47 – 0.24), p=0.52 | |
| Socialising with household members | Before lockdown | 1.59 (1.67) | 1.26 (1.55) | 1.08 (1.16) | 1.29 (1.48) | 1.57 (1.58) | 1.28 (1.53) | 1.29 (1.45) | 1.48 (1.77) |
|  | During lockdown | 1.69 (1.70) | 1.41 (1.43) | 1.42 (1.39) | 1.50 (1.50) | 1.64 (1.59) | 1.49 (1.71) | 1.49 (1.45) | 1.55 (1.89) |
|  | Mean difference (SD) | 0.05 (1.46) | 0.14 (1.23) | **0.32 (1.10)*** | **0.19 (1.24)*** | -0.02 (1.50) | 0.11 (1.43) | **0.19 (1.19)*** | 0.05 (1.86) |
|  | Results of ANOVA/ unpaired t-test comparing differences across groups | F(2, 603)=2.27, p=0.10 | | | 0.21 (-0.16 – 0.58), p=0.26 | |  | 0.14 (-0.20 – 0.48), p=0.42 | |
| Chores | Before lockdown | 0.68 (0.95) | 0.54 (0.71) | 0.50 (0.68) | 0.54 (0.75) | 0.78 (1.07) | 0.78 (0.89) | 0.52 (0.67) | 0.97 (1.27) |
|  | During lockdown | 0.86 (1.30) | 0.62 (0.80) | 0.58 (0.56) | 0.65 (0.90) | 1.07 (1.56) | 0.72 (0.64) | 0.60 (0.75) | 1.25 (1.65)1 |
|  | Mean difference (SD) | **0.10 (0.67)*** | **0.08 (0.62)*** | **0.08 (0.58)*** | **0.09 (0.55)*** | 0.16 (1.07) | -0.07 (0.72) | **0.08 (0.56)*** | **0.17 (0.74)*** |
|  | Results of ANOVA/ unpaired t-test comparing differences across groups | F(2,602)=0.06, p=0.94 | | | -0.07 (-0.25 – 0.10), p=0.42 | |  | -0.08 (-0.24 – 0.07), p=0.27 | |
| Sleep on weekdays | Before lockdown | 9.27 (1.03) | 9.14 (1.24) | 9.33 (1.00) | 9.32 (1.04) | 8.84 (1.18) | 8.83 (1.44) | 9.27 (1.08) | 9.02 (1.19) |
|  | During lockdown | 10.38 (1.57) | 10.04 (1.34) | 10.13 (1.18) | 10.14 (1.25) | 10.25 (2.02) | 10.54 (1.98) | 10.17 (1.33) | 10.28 (1.90) |
|  | Mean difference (SD) | **1.11 (1.62)*** | **0.86 (1.13)*** | **0.77 (0.99)*** | **0.82 (1.09)*** | **1.32 (1.79)*** | **1.59 (2.31)*** | **0.87 (1.25)*** | **1.26 (1.58)*** |
|  | Results of ANOVA/ unpaired t-test comparing differences across groups | **F(2, 583)=3.64, p=0.03** | | | **-0.50 (-0.85 - -0.15), p=0.005** | |  | **-0.39 (-0.74 - -0.04), p=0.03** | |
| Sleep on weekends | Before lockdown | 10.51 (1.26) | 10.19 (1.47) | 10.38 (1.31) | 10.40 (1.32) | 10.05 (1.53) | 10.24 (1.44) | 10.41 (1.34) | 9.85 (1.33) |
|  | During lockdown | 10.50 (1.69) | 10.29 (1.49) | 10.54 (1.47) | 10.42 (1.42) | 10.18 (1.93) | 11.00 (2.32) | 10.47 (1.52) | 10.25 (1.87) |
|  | Mean difference (SD) | 0.01 (1.36) | 0.1 (1.04) | **0.16 (1.37)*** | 0.03 (1.06) | 0.18 (1.62) | **0.75 (2.42)*** | 0.06 (1.23) | **0.43 (1.57)*** |
|  | Results of ANOVA/ unpaired t-test comparing differences across groups | F(2, 590)=0.78, p=0.46 | | | -0.14 (-0.48 – 0.19), p=0.39 | |  | **-0.37 (-0.71 - -0.03), p=0.03** | |
| Weekly exercise | Before lockdown | 2.81 (2.52) | 3.97 (3.00) | 4.35 (2.63) | 3.70 (2.82) | 3.37 (3.01) | 3.79 (2.23) | 3.72 (2.80) | 3.33 (2.83) |
|  | During lockdown | 3.08 (2.11) | 3.37 (2.04) | 3.96 (1.96) | 3.51 (2.04) | 2.74 (2.42) | 3.69 (1.80) | 3.49 (2.04) | 3.32 (2.32) |
|  | Mean difference (SD) | 0.32 (2.47) | **-0.60 (2.93)*** | **-0.39 (2.51)*** | -0.18 (2.72) | -0.57 (2.26) | -0.12 (2.37) | **-0.23 (2.67)*** | 0.03 (2.5) |
|  | Results of ANOVA/ unpaired t-test comparing differences across groups | **F(2, 618)=7.03, p=0.001** | | | 0.39 (-0.38 – 1.17), p=0.32 | |  | -0.26 (-0.94 – 0.43), p=0.46 | |

*paired t-test significant at p<0.05; Family Affluence Score (FAS); free school meals (FSM); analysis of variance (ANOVA)
