# Supplementary material for: Impact of the COVID-19 lockdown in the United Kingdom on adolescent’s time use (CONTRAST study)
Source: PLoS One. 2025 Jan 16;20(1):e0310597. doi: 10.1371/journal.pone.0310597 (PMC11737780; doi:10.1371/journal.pone.0310597)
Supplement: S4 Table — (DOCX) [file pone.0310597.s004.docx]

**Impact of the COVID-19 lockdown in the United Kingdom on adolescent’s time use (CONTRAST study)**

I.Pokhilenko,^1^ E. Frew,^1^ M. Murphy,^2^ M. Pallan^2^

^1^Centre for Economics of Obesity, Institute of Applied Health Research, University of Birmingham

^2^Institute of Applied Health Research, University of Birmingham

## **S4 Table. Changes in the frequency of academic and non-academic tuition and results of Wilcoxon signed rank test (z-scores)**

|  | | FAS | | | FSM eligible | | | Food insecurity | |
| --- | --- | --- | --- | --- | --- | --- | --- | --- | --- |
|  |  | 1 | 2 | 3 | No | Yes | Don’t know | No | Yes |
| Academic tuition | Same or decrease  N (%) | 205 (94%) | 185 (93%) | 188 (92%) | 487 (93%) | 51 (98%) | 42 (91%) | 511 (93%) | 59 (94%) |
|  | Increase  N (%) | 13 (6%) | 13 (7%) | 17 (8%) | 39 (7%) | 1 (2%) | 4 (9%) | 40 (7%) | 4 (6%) |
|  | Results of Wilcoxon signed rank test (z-scores) | **-2.14*** | -0.81 | -0.45 | **-2.27*** | 1.03 | -0.34 | -1.92 | -0.78 |
| Non-academic tuition | Same or decrease  N (%) | 189 (87%) | 165 (84%) | 171 (83%) | 443 (85%) | 45 (88%) | 40 (87%) | 464 (85%) | 54 (86%) |
|  | Increase  N (%) | 28 (13%) | 31 (16%) | 34 (17%) | 81 (15%) | 6 (12%) | 6 (13%) | 84 (15%) | 9 (14%) |
|  | Results of Wilcoxon signed rank test (z-scores) | **-3.7*** | **-3.66*** | **-3.52*** | **-5.85*** | -1.353 | -1.879 | **-6.07*** | -2.03 |

*significant at p<0.05; Family Affluence Scale (FAS); free school meals (FSM)
