# Supplementary material for: Impact of the COVID-19 lockdown in the United Kingdom on adolescent’s time use (CONTRAST study)
Source: PLoS One. 2025 Jan 16;20(1):e0310597. doi: 10.1371/journal.pone.0310597 (PMC11737780; doi:10.1371/journal.pone.0310597)
Supplement: S5 Table — (DOCX) [file pone.0310597.s005.docx]

**Impact of the COVID-19 lockdown in the United Kingdom on adolescent’s time use (CONTRAST study)**

I.Pokhilenko,^1^ E. Frew,^1^ M. Murphy,^2^ M. Pallan^2^

^1^Centre for Economics of Obesity, Institute of Applied Health Research, University of Birmingham

^2^Institute of Applied Health Research, University of Birmingham

## **S5 Table. Results of the Tukey post hoc tests testing the significance of time use changes across family affluence score groups**

| Category of time use | Comparison groups | Contrast (95% CI) | P value |
| --- | --- | --- | --- |
| School work | FAS group 2 vs 1 | **0.76 (0.13 – 1.39)** | **0.01** |
|  | FAS group 3 vs 1 | **0.91 (0.29 – 1.54)** | **0.002** |
|  | FAS group 3 vs 2 | 0.16 (-0.48 – 0.79) | 0.84 |
| Sleep on weekdays | FAS group 2 vs 1 | -0.25 (-0.55 – 0.06) | 0.14 |
|  | FAS group 3 vs 1 | **-0.33 (-0.63 – -0.03)** | **0.03** |
|  | FAS group 3 vs 2 | -0.09 (-0.39 – 0.22) | 0.79 |
| Weekly exercise | FAS group 2 vs 1 | **-0.92 (-1.53 - -0.31)** | **0.001** |
|  | FAS group 3 vs 1 | **-0.71 (-1.31 - -0.11)** | **0.02** |
|  | FAS group 3 vs 2 | 0.21 (-0.41 – 0.82) | 0.71 |

Family Affluence Score (FAS); confidence interval (CI)
