# Supplementary material for: Impact of the COVID-19 lockdown in the United Kingdom on adolescent’s time use (CONTRAST study)
Source: PLoS One. 2025 Jan 16;20(1):e0310597. doi: 10.1371/journal.pone.0310597 (PMC11737780; doi:10.1371/journal.pone.0310597)
Supplement: S6 Table — (DOCX) [file pone.0310597.s006.docx]

**Impact of the COVID-19 lockdown in the United Kingdom on adolescent’s time use (CONTRAST study)**

I.Pokhilenko,^1^ E. Frew,^1^ M. Murphy,^2^ M. Pallan^2^

^1^Centre for Economics of Obesity, Institute of Applied Health Research, University of Birmingham

^2^Institute of Applied Health Research, University of Birmingham

## **S6 Table. Results of the regression analysis of changes in time use**

| Category of time use | Socioeconomic indicators | | | | | Demographic characteristics | | | | | | | | |
| --- | --- | --- | --- | --- | --- | --- | --- | --- | --- | --- | --- | --- | --- | --- |
|  | FAS group (1 – reference category) | | Free school meal eligibility (Not eligible – reference category) | | Experience of food insecurity (No – reference category) | Gender (Male – reference) | Age | Ethnicity (White – reference) | | | Country of residence (England – reference) | Language spoken at home (English/Welsh – reference) | School type (State – reference) | |
|  | 2 | 3 | Eligible | Don’t know | Yes | Female |  | Asian/Asian British | Black/ African/Caribbean/ Black British | Other ethnic group | Scotland/Wales | Other | Grammar | Private |
| School work (total) | 0.30 | -0.03 | n/a | n/a | n/a | 0.18 | -0.00 | -0.80 | -0.39 | -0.82 | -0.34 | 0.10 | 0.57* | 3.43* |
|  | n/a | n/a | -0.61 | -0.09 | n/a | 0.18 | -0.00 | -0.73 | 0.41 | -0.93* | -0.36 | 0.02 | 0.54 | 3.40* |
|  | n/a | n/a | n/a | n/a | -0.33 | 0.19 | -0.02 | -0.71 | 0.32 | -1.05* | -0.45 | -0.01 | 0.57* | 3.33* |
| Activities outside of schoolwork that help me learn new knowledge and skills | -0.17 | 0.19 | n/a | n/a | n/a | -0.16 | 0.07 | 0.57* | 0.11 | 0.34 | -0.03 | -0.05 | 0.00 | 0.01 |
|  | n/a | n/a | 0.03 | 0.29 | n/a | -0.17 | 0.07 | 0.54 | 0.09 | 0.42 | -0.06 | 0.00 | 0.02 | 0.06 |
|  | n/a | n/a | n/a | n/a | -0.05 | -0.21 | 0.07 | 0.55 | -0.03 | 0.51 | -0.03 | 0.02 | 0.06 | 0.08 |
| Reading for fun | 0.02 | 0.15 | n/a | n/a | n/a | -0.06 | 0.08* | -0.14 | 0.38* | -0.17 | -0.09 | -0.05 | -0.09 | -0.05 |
|  | n/a | n/a | -0.11 | 0.36* | n/a | -0.07 | 0.07* | -0.16 | 0.35* | -0.15 | -0.13 | -0.04 | -0.08 | -0.03 |
|  | n/a | n/a | n/a | n/a | 0.01 | -0.08 | 0.07* | -0.17 | 0.36* | -0.15 | -0.08 | -0.03 | -0.06 | 0.01 |
| Screen time (total) | 0.28 | 0.36 | n/a | n/a | n/a | 0.13 | 0.23 | 0.47 | 0.77 | 0.50 | -0.40 | -0.51 | -0.50 | 0.13 |
|  | n/a | n/a | 0.45 | -0.48 | n/a | 0.12 | 0.23 | 0.38 | 0.61 | 0.43 | -0.29 | -0.53 | -0.36 | 0.27 |
|  | n/a | n/a | n/a | n/a | 0.64 | 0.21 | 0.12 | 0.29 | 0.56 | 0.49 | -0.29 | -0.56 | -0.34 | 0.24 |
| Spending time chatting with friends on social media | 0.00 | -0.03 | n/a | n/a | n/a | 0.21 | 0.15* | -0.15 | 0.15 | 0.33 | -0.09 | -0.13 | -0.11 | 0.38* |
|  | n/a | n/a | 0.33 | -0.03 | n/a | 0.21 | 0.15* | -0.16 | 0.15 | 0.28 | -0.07 | -0.15 | -0.09 | 0.39* |
|  | n/a | n/a | n/a | n/a | 0.20 | 0.19 | 0.13* | -0.17 | 0.17 | 0.35 | -0.12 | -0.07 | -0.08 | 0.38* |
| Watching TV/ Netflix/ YouTube/TikTok etc. | 0.22 | 0.22 | n/a | n/a | n/a | 0.35* | 0.08 | -0.03 | 0.74 | -0.06 | -0.37 | -0.08 | -0.27 | -0.21 |
|  | n/a | n/a | -0.26 | -0.19 | n/a | 0.34* | 0.09 | -0.05 | 0.65 | -0.05 | -0.34 | -0.07 | -0.23 | -0.14 |
|  | n/a | n/a | n/a | n/a | 0.16 | 0.35* | 0.09 | -0.05 | 0.59 | -0.10 | -0.35 | -0.12 | -0.21 | -0.15 |
| Playing games on a device | 0.02 | 0.14 | n/a | n/a | n/a | -0.44* | -0.01 | 0.63* | -0.19 | 0.23 | 0.10 | -0.25 | -0.08 | -0.08 |
|  | n/a | n/a | 0.37 | -0.24 | n/a | -0.44* | -0.01 | 0.59* | -0.24 | 0.20 | 0.16 | -0.25 | -0.03 | -0.02 |
|  | n/a | n/a | n/a | n/a | 0.17 | -0.42* | -0.01 | 0.50 | -0.24 | 0.24 | 0.16 | -0.26 | -0.04 | -0.03 |
| Socialising with household members | 0.14 | 0.34* | n/a | n/a | n/a | 0.11 | -0.00 | 0.15 | 0.05 | -0.39 | -0.07 | -0.06 | -0.20 | -0.29 |
|  | n/a | n/a | -0.22 | -0.09 | n/a | 0.10 | 0.01 | 0.09 | -0.08 | -0.33 | -0.05 | -0.01 | -0.15 | -0.19 |
|  | n/a | n/a | n/a | n/a | -0.17 | 0.11 | 0.02 | 0.08 | -0.04 | -0.36 | -0.05 | -0.02 | -0.15 | -0.14 |
| Chores | -0.03 | -0.03 | n/a | n/a | n/a | 0.07 | 0.05* | 0.12 | -0.33* | 0.02 | 0.01 | -0.02 | -0.06 | 0.01 |
|  | n/a | n/a | 0.08 | -0.16 | n/a | 0.08 | 0.05* | 0.14 | -0.32* | -0.01 | 0.02 | -0.04 | -0.08 | 0.01 |
|  | n/a | n/a | n/a | n/a | 0.03 | 0.07 | 0.06* | 0.11 | -0.19 | -0.01 | -0.00 | -0.03 | -0.08 | 0.05 |
| Exercise | -0.80* | -0.53 | n/a | n/a | n/a | 0.12 | 0.24* | 0.59 | -0.60 | -0.59 | -0.54 | 0.53 | 0.47 | -0.93* |
|  | n/a | n/a | -0.57 | 0.07 | n/a | 0.15 | 0.25* | 0.74 | -0.29 | -0.42 | -0.64 | 0.62 | 0.26 | -1.12* |
|  | n/a | n/a | n/a | n/a | 0.88 | 0.12 | 0.23* | 0.70 | -0.22 | -0.46 | -0.45 | 0.64 | 0.28 | -1.10* |
| Sleep during weekdays | -0.22 | -0.16 | n/a | n/a | n/a | -0.00 | 0.13* | 0.72* | 0.14 | 0.31 | 0.03 | -0.34 | -0.19 | -0.41* |
|  | n/a | n/a | 0.40 | 0.76* | n/a | -0.01 | 0.11* | 0.77* | 0.27 | 0.23 | -0.05 | -0.41* | -0.18 | -0.45* |
|  | n/a | n/a | n/a | n/a | 0.30 | -0.01 | 0.12* | 0.78* | 0.26 | 0.26 | 0.04 | -0.39* | -0.20 | -0.44* |
| Sleep during weekends | 0.16 | 0.23 | n/a | n/a | n/a | -0.07 | -0.01 | 0.61* | 0.11 | 0.14 | -0.33 | -0.04 | -0.18 | -0.11 |
|  | n/a | n/a | 0.19 | 0.75* | n/a | -0.07 | -0.02 | 0.50* | -0.01 | 0.19 | -0.14 | -0.30 | -0.11 | -0.06 |
|  | n/a | n/a | n/a | n/a | 0.44* | -0.09 | -0.01 | 0.51* | -0.01 | 0.22 | -0.01 | -0.32 | -0.09 | -0.02 |
| Academic tuition | 1.56 | 1.88 | n/a | n/a | n/a | 0.98 | 1.02 | 2.00 | 1.43 | 1.51 | 0.81 | 1.46 | 0.75 | 0.45 |
|  | n/a | n/a | 0.18 | 1.13 | n/a | 1.00 | 1.07 | 1.72 | 1.04 | 1.99 | 0.77 | 1.72 | 0.73 | 0.49 |
|  | n/a | n/a | n/a | n/a | 0.73 | 1.00 | 1.05 | 1.61 | 1.08 | 1.80 | 0.87 | 1.69 | 0.82 | 0.53 |
| Non-academic tuition | 0.95 | 0.94 | n/a | n/a | n/a | 1.13 | 1.03 | 0.65 | 0.20 | 1.13 | 0.65 | 1.88 | 1.47 | 2.12* |
|  | n/a | n/a | 0.64 | 0.78 | n/a | 1.13 | 1.04 | 0.69 | 0.21 | 1.08 | 0.64 | 1.86 | 1.38 | 2.07* |
|  | n/a | n/a | n/a | n/a | 0.86 | 1.12 | 1.04 | 0.68 | 0.22 | 1.08 | 0.68 | 1.84 | 1.43 | 2.09* |
