# Supplementary material for: Impact of the COVID-19 lockdown in the United Kingdom on adolescent’s time use (CONTRAST study)
Source: PLoS One. 2025 Jan 16;20(1):e0310597. doi: 10.1371/journal.pone.0310597 (PMC11737780; doi:10.1371/journal.pone.0310597)
Supplement: Attachment — Submitted filename: review v1.docx [file pone.0310597.s007.docx]

| Date received | 12/4/24 |
| --- | --- |
| Journal | PLoSOne |
| Title | Impact of the COVID-19 lockdown in the United Kingdom on adolescent’s time use (CONTRAST study) |
| MS | PONE-D-23-40253 |
| funded | his study has received funding from the UKRI Economic and Social Research Council Impact Acceleration Account programme. |
| Data available | The data associated with this manuscript are available on Zenodo via 10.5281/zenodo.10245892. |

| Page | Comment |
| --- | --- |
| Abs | Sound abstract containing main research questions and outcomes |
| intro |  |
| Methods  12  13  Line 120  14  Line 124  16  Line 143 | Where/location was the study completed. were the adolescents from 1 high school for example? How many schools age -range type and so forth.  What was the rational for creating tertile groups from FAS scores, why not use as a continuous variable? This measure partially corresponds… to what degree and .. the relevance?? So hardship is important but it is unclear how valid the measures were given that they “partially” correspond.  Was the allocation of time questions section validated elsewhere?  For example, respondents who reported spending  between 0 and 30 minutes per day on a given activity were assigned the value of 0.25, corresponding  to 15 minutes or one quarter of an hour. So a participant may do ) but receive 15 mins??  There are many assumptions being made in time allocation.. the change in perceived time may be valid but the absolute numbers are less robust in real terms.  What is the value of FAS+FSM+FI??? In answering the research question.. it seems that this paper should focus on FAS and thus include more of the data included in the supplementary section in the main paper. |
| Results  17  Line 186  Table 2  Page 19  Line 199  Descriptive analysis  P20  Line 231  P22  Table 5 | So while the aim was to investigate affluence, COVID lockdown and behaviors the sample is significantly biased as stated in section 3.1  Is ethnic/school type/FSM etc breakdown reflective of the population. There are very small numbers in FSM and don’t know as well as those who have food insecurity?  Were the data able to split for school work using screens compared to recreational screen use?  Table 2 needs units of measurement.  Table S3?? State supplementary  Given the main research question it would be better to have the descriptive split into the sub groups being used for analysis, particularly the FSM/FAS/gender etc groupings.  Physical activity should be used to replace the term exercise. Check out the differences in the definition for physical activity and exercise. (here nd throughout the paper).  How relevant is the analysis for model 2 given that “don’t know” is unknown so maybe eligible or not?? Likewise the numbers of children who have food insecurity is very small. Model 1 is based on a balanced dataset for FAS at least.  There are relatively few significant results in the table. Surprising given that very few of the Cis cross 0. |
| Discussion  P23  Line 280  Line 287  Line 300  P24  Line 301  Line 302  P25  Line 319  P26  Line 334  P27  Lines 377-387  P28 lines 408-417 | The first section of the discussion reads a little more like a results section. Where does the scientific literature sit in relation to these results?  Type of school emerges here as a main issue. But these may be collinear with “FAS.”  So except for sleep, exercise, socilaising with household inequalities lessened between groups during lockdown?  Could your screen time data be split into screentime for learning and screen time for recreation/socializing eg gaming social media.  Exercise v physical activity?? Check definition  What is a “slight” non significant increase?  Do more less affluent children participate less in organized sport because of its cost or are there other factors at play such as the built environment, accessibility and so forth.  Sleep is a crucial behaviour and you briefly mention sleep quality did you also think about sleep efficiency?  I agree with your self-critical view about the use of the FAS, FSM and FI measures in that they all seem surrogates for deprivation yet maybe measuring different things. Further they are relatively blunt instruments. Did you consider generating a latent measure by combining data from the three different sets of deprivation data? You allude to this in lines 352-354.  Other limitations are noted but given the title and hypothesis for this study the biased sample somewhat invalidates the research question. One of the interesting academic issues the study brings up is the use of the 3 different tools of FAS, FSM and FI particularly those repoted in lines 392-393  This section is very interesting but I am left wondering about the evidence from the empirical work in the study that contributes towards cognitive learning and social capital for example?  This is an excellent well written critical section |
